# Combined femoral and sciatic nerve block versus femoral and local infiltration anesthesia for pain control after total knee arthroplasty: a meta-analysis of randomized controlled trials

**DOI:** 10.1186/s13018-016-0495-6

**Published:** 2016-12-07

**Authors:** Jian Li, Xinlian Deng, Tao Jiang

**Affiliations:** 1 Department of Pain, Yidu Central Hospital of Weifang, Qingzhou, Shangdong Province China; 2 Department of Orthopaedics, The Second Affiliated Hospital of Zhejiang Chinese Medical University, 318 Chaowang Road, Hangzhou, Zhejiang 310005 China

**Keywords:** Sciatic nerve block, Local infiltration anesthesia, Total knee replacement, Meta-analysis

## Abstract

**Background:**

The purpose of this systematic review and meta-analysis of randomized controlled trials (RCTs) was to evaluate the effect of combined femoral and sciatic nerve block (SNB) versus femoral and local infiltration anesthesia (LIA) after total knee arthroplasty (TKA).

**Methods:**

The electronic databases PubMed, Embase, Cochrane Library, and Web of Science were searched from their inception to 15 June 2016. Articles comparing combined femoral and SNB versus femoral and LIA for pain control were eligible for this meta-analysis. This systematic review and meta-analysis was performed according to the PRISMA statement criteria. The primary endpoint was the visual analogue scale (VAS) score with rest at 12, 24, and 48 h, which represents the pain control after TKA. Data regarding active knee flexion, length of hospital stay, anesthesia time, and morphine use at 24 and 48 h were also compiled. The complications of postoperative nausea and vomiting (PONV) and fall were also noted to assess the safety of morphine-sparing effects. After testing for publication bias and heterogeneity across studies, the data were aggregated for random-effects modeling when necessary.

**Results:**

Seven clinical trials with 615 patients were included in the meta-analysis. The pooled results indicated that SNB was associated with a lower VAS score at 12 h (MD = −6.96; 95% CI −8.36 to −5.56; *P* < 0.001) and 48 h (MD = −2.41; 95% CI −3.90 to −0.91; *P* < 0.001) after TKA. There was no significant difference between the SNB group and the LIA group in terms of the VAS score at 24 h (MD = 0.67; 95% CI −0.31 to 1.66; *P* = 0.182). The anesthesia time in the LIA group was shorter than in the SNB group, and the difference was statistically significant (MD = 4.31, 95% CI 1.34 to 7.28, *P* = 0.004). There were no significant differences between the groups in terms of active knee flexion, length of hospital stay, morphine use, PONV, and the occurrence of falls.

**Conclusions:**

SNB may provide earlier anesthesia effects than LIA when combined femoral nerve block (FNB); however, there were no differences in morphine use, active knee flexion, and PONV between the groups. The LIA group spent less time under anesthesia, suggesting that LIA may offer a practical and potentially safer alternative to SNB.

**Electronic supplementary material:**

The online version of this article (doi:10.1186/s13018-016-0495-6) contains supplementary material, which is available to authorized users.

## Background

Total knee arthroplasty (TKA) is a common procedure for improving mobility and quality of life in patients with osteoarthritis or rheumatoid arthritis. However, TKA itself is always associated with moderate to severe pain after surgery. It is reported that 60 and 30% of TKA patients experience severe and moderate pain, respectively [1]. Currently, both femoral nerve block (FNB) and local infiltration anesthesia (LIA) can provide effective analgesia, facilitate early mobilization, and reduce the length of hospital stay [2, 3]. Chan et al. [4] conducted a meta-analysis to compare FNB with other analgesic techniques, and the results indicated that there was insufficient data to draw a definitive conclusion regarding FNB with LIA. Although FNB is a well-accepted and commonly used technique for regional anesthesia after TKA, previous studies indicate that some patients experience significant postoperative pain despite the administration of FNB [5, 6]. Compared with peripheral nerve block, LIA is an alternative, convenient anesthetic technique that is usually performed by orthopedic surgeons. Meanwhile, the efficacy and safety of LIA is comparable to that of epidural anesthesia, FNB, and intrathecal morphine [7]. Therefore, anesthesia via sciatic nerve block (SNB) and LIA are two major options for supplementing FNB to relieve pain after TKA [8–10]. However, there is no consensus regarding which anesthesia method is preferable to relieve pain as an adjunct to FNB. Thus, a meta-analysis of randomized controlled trials (RCTs) was conducted to compare the efficacy and safety of pain control with SNB versus LIA when combined with FNB after TKA.

## Methods

This review is registered in Protocol registration: PROSPERO 2016 CRD42016050735.

### Search strategy

The electronic databases PubMed, Embase, Cochrane Library, and Web of Science were searched from their inception to 15 June 2016. The search terms included local infiltration anesthesia, femoral nerve block, sciatic nerve block, and total knee arthroplasty. The Boolean operators “AND” and “OR” were used to couple these terms. The details of the search strategy are displayed in Additional file 1. There were no restrictions regarding language and publication date. We also manually retrieved reference lists from the identified studies and relevant review studies to identify additional relevant studies. Two investigators independently assessed the titles and abstracts of the studies identified by the retrieval. The full text of the remaining studies was then reviewed to ensure that they met the eligibility criteria. Disagreements were settled by consulting a third reviewer.

### Inclusion criteria and study selection

All RCTs comparing combined femoral and SNB versus femoral and LIA for pain control were eligible for this meta-analysis. If there were more than one eligible trials from one team, the study with most recent publication data were enrolled for analysis. Studies that included bilateral TKA, revision of TKA, or other anesthetic methods were excluded. All non-randomized trials were also excluded.

### Data abstraction

Two reviewers extracted the data independently using a predefined data extraction form. Disagreements were resolved through discussion or consensus with a third reviewer. The data extracted included the first author, publication year, study characteristics (number of patients and percent of female patients), participant characteristics (i.e., mean age, type of anesthesia, operative approach, and type of prosthesis), and the length of follow-up. For studies with insufficient information, the reviewers tried to contact the first author via e-mail or telephone to obtain the original data. After duplicates were excluded, two reviewers independently read the titles and abstracts of the selected literature. Most of the articles were excluded based on the topic of the article provided in the title or abstract, and disagreements regarding whether an article should be included were resolved via discussion or consultation with a senior reviewer. Postoperative pain intensity was measured using a 100-point visual analogue scale (VAS). The 10-point VAS score was converted to a 100-point VAS score. Data in other forms (i.e., median, interquartile range, and mean ± 95% confidence interval (CI)) were converted to mean ± SD as described in the Cochrane handbook [11]. If the data were not reported numerically, we extracted them from the published figures using the “GetData Graph Digitizer” software [12].

### Quality assessment

Two independent reviewers assessed the methodological quality of the included trials according to the Cochrane Collaboration recommendations [11]. The following information was evaluated: random sequence generation, allocation concealment, blinding of outcome assessments, incomplete outcome data, selective reporting, and other biases. An independent arbiter was consulted to reconcile any disagreement.

### Statistical analysis

Continuous outcomes, such as the VAS at 12, 24, and 48 h, morphine consumption at 24 and 48 h, active knee flexion, the length of hospital stay, and anesthesia time, were expressed as the mean difference (MD) with the respective 95% CIs. Discontinuous outcomes (the rate of postoperative nausea and vomiting (PONV) and fall) were expressed as the relative risk (RR) with 95% CIs. Statistical significance was set at *P* < 0.05 to summarize the findings across the trials. Stata 12.0 software (Stata Corp., College Station, TX) was used for the meta-analysis. Statistical heterogeneity was tested using the I^2^ statistic. A value of I^2^ > 50% was considered to indicate statistical heterogeneity, and a random effects model was applied. Then, sensitivity analysis was conducted to identify potential sources of heterogeneity. When there was no statistical evidence of heterogeneity, a fixed-effects model was adopted. A subgroup analysis was conducted to identify whether the type of FNB (continuous FNB versus single-shot FNB) and anesthesia (general anesthesia versus spinal anesthesia) affected the VAS at 12, 24, and 48 h.

### Trial sequential analysis

Because cumulative meta-analyses carry a risk of producing random errors, mainly because of sparse data and repetitive testing of cumulative data [13–15], trial sequential analysis was performed in case the data were too sparse to draw firm conclusions. Trial sequential analysis is comparable to interim analysis in a single trial, and the trial sequential monitoring boundary can be applied to meta-analysis to determine whether the *P* value is small enough to show the anticipated effect and whether the trial should be terminated early [16]. If the trial sequential analysis boundary or the futility zone is crossed, more trials are unnecessary.

## Results

### Search results and quality assessment

**Fig. 1:**
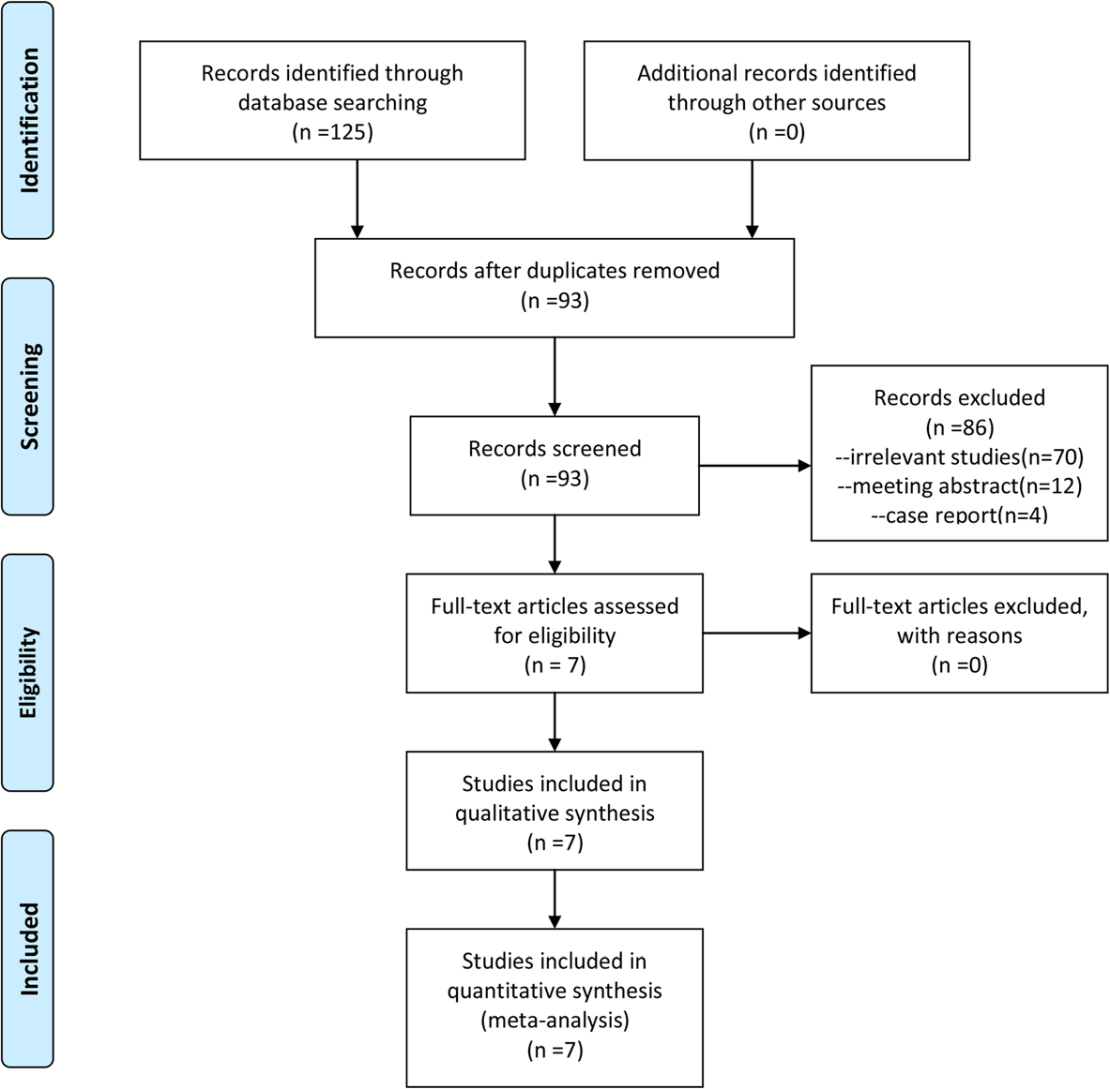
The flow diagram of the included studies. *RCT* randomized clinical trial

In the initial search, 125 potentially relevant trials were identified, and 32 duplications were removed using the Endnote X7 software (Thomson Research Soft Company, America). The titles and abstracts of 93 articles were reviewed to determine whether they met the inclusion criteria; the search details are shown in Fig. 1. During the search process, a total of three disagreements occurred and were resolved by reading the full-length of article and conferring with the senior author. Finally, only seven RCTs involving 615 patients were included for meta-analysis [9, 17–22]. The number of patients in the SNB group and the LIA group were 307 and 308, respectively. The baseline data of the included studies were comparable, and all the patients in the included studies were prepared for primary unilateral TKA (Additional file 2). Four studies [17, 19, 21, 23] performed continuous FNB, and the remaining three studies [18, 20, 22] performed single-shot FNB. Two studies administered intraoperative anesthesia in spinal anesthesia and the rest studies in general anesthesia. Six studies perform the FNB with nerve stimulator [9, 17, 18, 20–22] and four studies with ultrasound devices [18–20, 22]. All studies administered with oral NSAIDs for postoperative anesthesia. Two studies administered acetaminophen [9, 22], two studies administered with diclofenac [18, 20], two studies with loxoprofen sodium [19, 21], and the rest one study [24] administered with celecoxib. The general characteristic can be seen in Table 1.

**Table 1:** The general characteristic of the included studies

Author and year	No of patients (SNB:LIA)	Gender male/female	Age (mean, SNB:LIA)	Anesthesia	Surgery method	FNB	The drug and doses of SNB	The drug and doses of LIA	Concomitant pain management
Mahadevan, D 2012	26/26	23/29	67.2/67.5	General anesthesia	Standard medial parapatellar	20 mL of 0.375% levobupivacaine	20 mL of 0.375% levobupivacaine	20 mL of 0.375% levobupivacaine	PCA for 48 h
Safa, B 2014	33/32	32/33	61.2/60.7	Spinal anesthesia	NS	20 mL of 0.5% ropivacaine	20 mL of 0.5% ropivacaine	50 mL of 0.2% ropivacaine	PCA for 48 h
Tanikawa, H 2014	23/23	7/39	72/71	General anesthesia	Standard mid-vastus approach	20 ml of 0.375% ropivacaine	20 ml of 0.375% ropivacaine	200 mg of ropivacaine and 0.5 ml of adrenaline	0.2% ropivacaine infusion
Gi, E 2014	24/25	4/45	78/77	General anesthesia	NS	20 ml 0.375% ropivacaine	20 ml 0.375% ropivacaine	60 ml 0.5% ropivacaine with 0.3 mg epinephrine	60 mg oral loxoprofen sodium every 8 h
Nagafuchi, M 2015	17/16	5/28	72/73	General anesthesia	Midvastus approach	20 mL of 0.375% ropivacaine	20 mL of 0.375% ropivacaine	100 mL of 0.2% ropivacaine by adding 0.5 mL of adrenaline	Continuous femoral block of 0.2% ropivacaine
Uesugi, K 2014	105/105	41/159	76.3/76	Spinal anesthesia	Themid-vastus approach	20 mL of 0.75% ropivacaine	10 mL of 0.75% ropivacaine	20 ml of 0.75% ropivacaine, physiological saline 20 mL, adrenaline 0.3 mg, morphine hydrochloride and dexamethasone 3.3 mg	NS
Spangehl, M. J 2015	79/81	71/89	67.8/67.7	General anesthesia	Medial parapatellar approach	30 mL 0.5% ropivacaine	30 mL 0.5% ropivacaine	Ropivacaine Epinephrine Ketorolac Morphine sulfate	Analgesic medications

*NS* not stated, *SNB* sciatic nerve block, *LIA* local infiltration anesthesia, *FNB* femoral nerve block, *PCA* patient controlled anesthesia

The quality assessment was as follows: three studies’ random sequence generation methods were not clear, and allocation concealment was performed in all the included studies. The other biases are shown in Table 2.

**Table 2:** The quality assessment of the included studies

Author and year	Random sequence generation	Allocation concealment	Blinding	Incomplete outcome data	Selective reporting	Other sources of bias
Mahadevan, D 2012 [9]	Unclear	Opaque sealed envelopes	Yes	Yes	No	No
Safa, B 2014 [17]	Computer generated	Hospital investigational pharmacy	Yes	Yes	No	No
Tanikawa, H 2014 [18]	Unclear	Sealed envelopes	Yes	Yes	No	No
Gi, E 2014 [19]	SPSS	Sealed envelopes	Yes	Yes	No	No
Nagafuchi, M 2015 [20]	Randomization web site	Storing the treatment allocation	Yes	Yes	No	No
Uesugi, K 2014 [21]	Unclear	Opaque envelope	Yes	Yes	Unclear	Unclear
Spangehl, M. J 2015 [22]	Computerized random number tables	Opaque envelope	Yes	Yes	No	No

### VAS score with rest at 12, 24, and 48 h

**Fig. 2:**
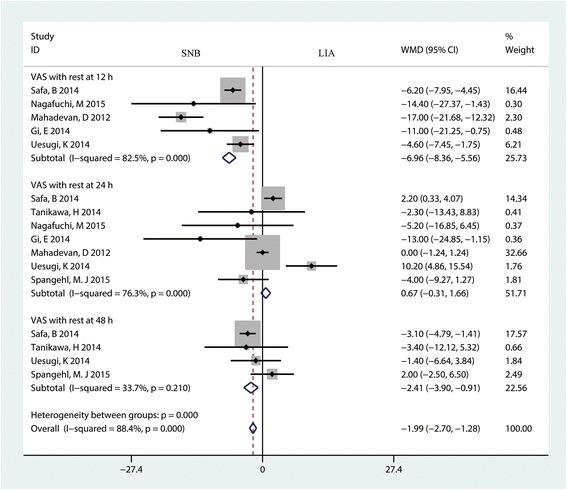
The forest plot comparing SNB and LIA for VAS score after TKA. An inverse variance fixed-effects model was used. Mean differences with 95% CIs are reported. *WMD* weighted mean difference

Five trials with 409 patients reported the VAS score with rest at 12 h for the SNB group and the LIA group. The pooled results indicated that SNB was associated with a lower VAS score at 12 h after TKA (MD = −6.96; 95% CI −8.36 to −5.56; *P* < 0.001, Fig. 2) and high heterogeneity (*P* = 0.000, I^2^ = 82.5%). Seven studies with 615 patients reported the VAS scores with rest at 24 h in the SNB group and the LIA group; the meta-analysis results indicated that there was no significant difference between the SNB group and the LIA group in terms of the VAS score at 24 h (MD = 0.67; 95% CI −0.31 to 1.66; *P* = 0.182, Fig. 2). Four studies with 481 patients were included in a meta-analysis that indicated that SNB can decrease VAS with rest at 48 h by a mean of 2.41 mm; this result was statistically significant (MD = −2.41; 95% CI −3.90 to −0.91; *P* < 0.001, Fig. 2) and had low heterogeneity (*P* = 0.210, I^2^ = 33.7%).

**Fig. 3:**
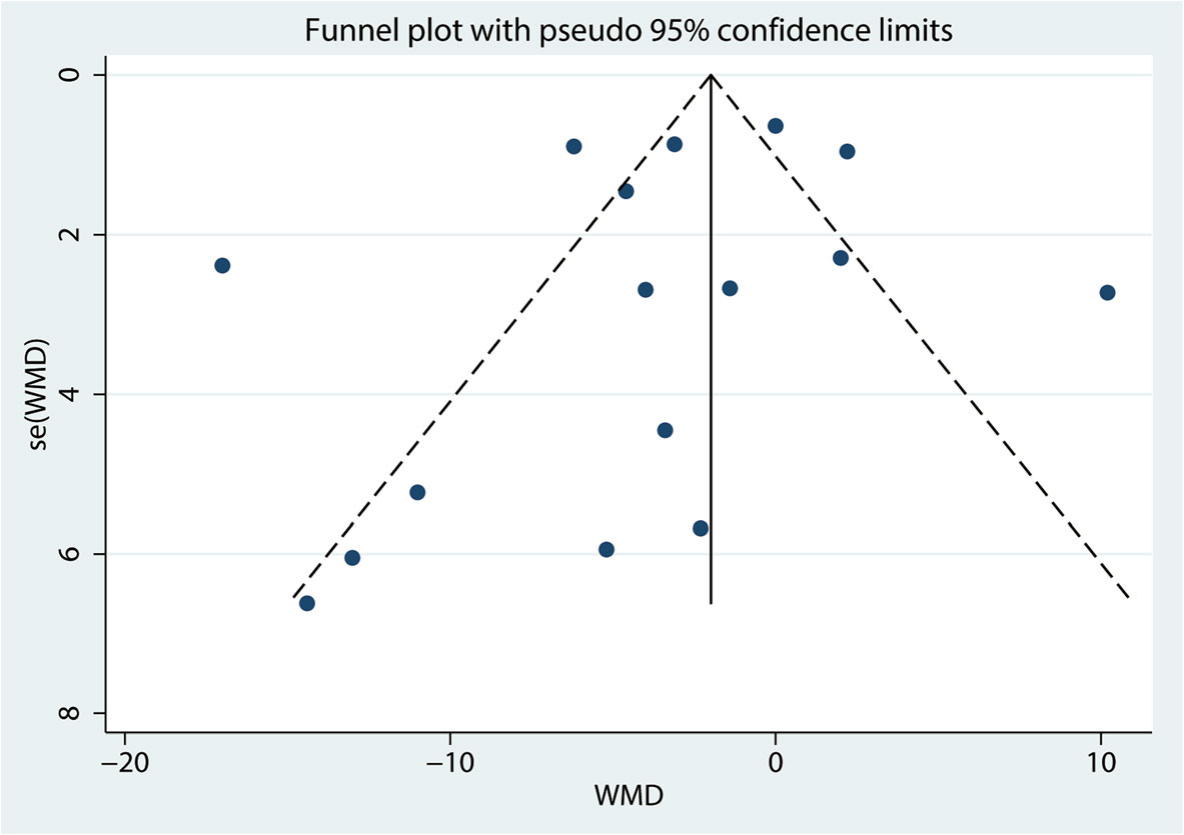
Funnel plot with pseudo 95% confidence limits

**Fig. 4:**
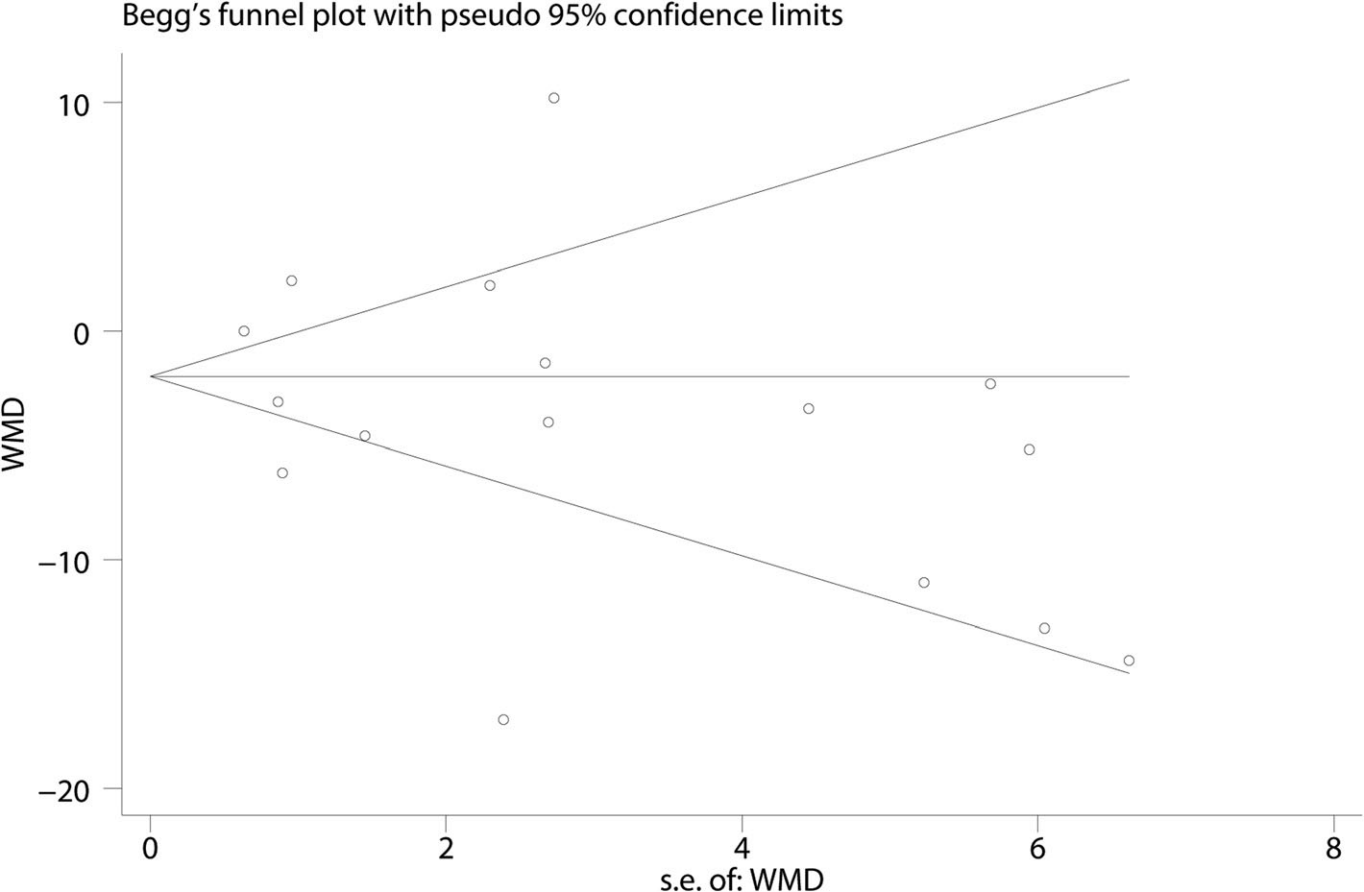
The publication bias between the studies, indicated by the funnel plot. *WMD* weighted mean difference

**Fig. 5:**
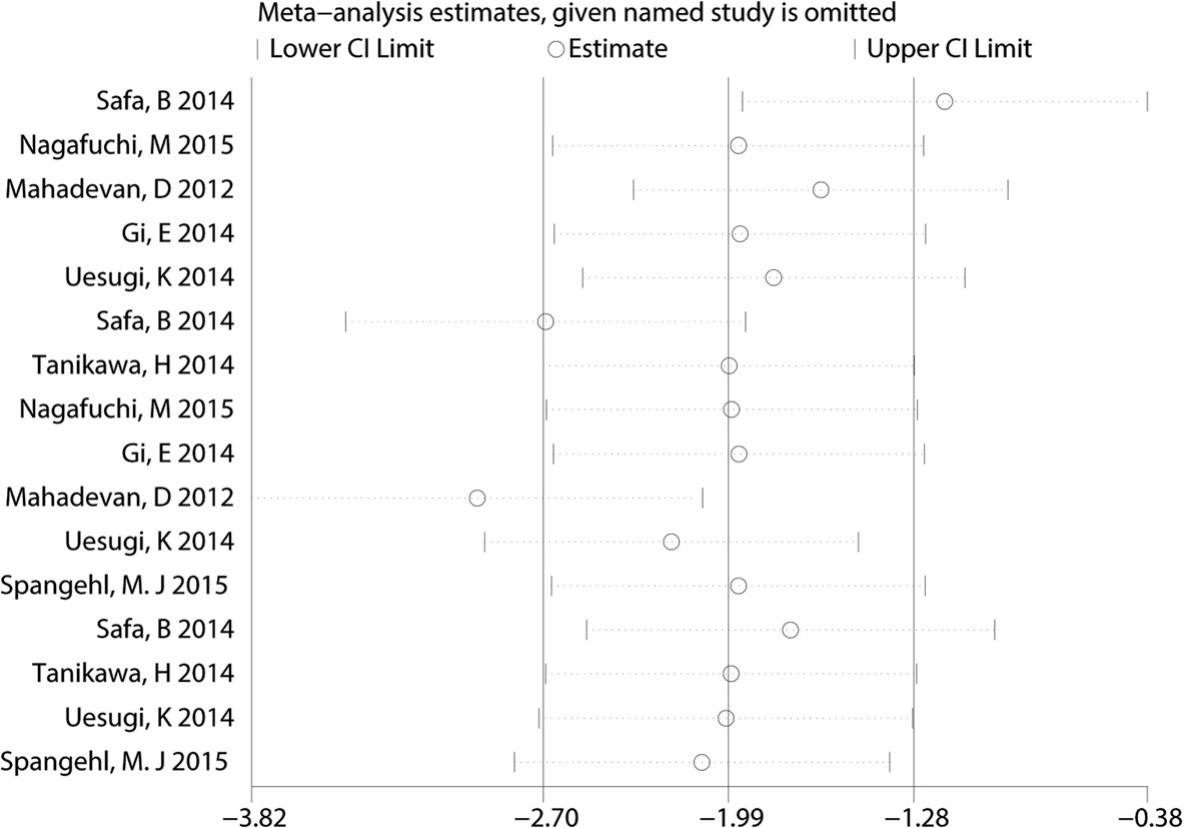
The result of Begg’s test for the VAS score after TKA

A funnel plot was then used to identify whether the studies showed publication bias, and the results indicated that there was no publication bias in the included studies (Fig. 3). Begg’s test further confirmed the results (*P* > 0.05, Fig. 4). Because there was a large degree of heterogeneity among the studies, a sensitivity analysis was performed to find the source of the heterogeneity. The results indicated that the study by Safa B may affect the final result (Fig. 5). We then excluded the data from the study by Safa B, and results are shown in Additional file 3. The subgroup analysis results are shown in Table 3. The SNB group had a lower VAS score with rest at 12 h when general anesthesia was administered compared with the LIA group. Furthermore, continuous FNB was associated with a lower VAS score with rest at 12 h than LIA was.

**Table 3:** Subgroup analysis for VAS with rest at 12, 24, and 48 h

Variables	Studies (*n*)	Patients (*n*)	*P* value	Incidence		
				MD (95% CI)	Heterogeneity *P* value (I^2^)	Model
VAS at 12 h						
SFNB	4	376	<0.001	−6.88 (−8.24,−5.47)	0.000 (86.1)	Random
CFNB	1	33	0.03	−14.4 (−27.37,−1.43)		
Spinal anesthesia	2	275	<0.001	−5.76 (−7.25,−4.27)	0.348 (0.0)	Fixed
General anesthesia	3	134	<0.001	−15.81 (−19.86,−11.77)	0.566 (0.0)	Fixed
VAS at 24 h						
SFNB	4	324	0.002	2.73 (0.98,4.47)	0.001 (86.3)	Random
CFNB	3	239	0.083	−3.91 (−8.32,0.51)	0.938 (0.0)	Fixed
Spinal anesthesia	2	275	0.145	5.79 (−2.01,13.59)	0.006 (87.0)	Random
General anesthesia	5	288	0.148	−2.84 (−6.69,1.01)	0.121 (45.2)	Fixed
VAS at 48 h						
SFNB	2	275	0.000	−2.94 (−4.55,−1.33)	0.545 (0.0)	Fixed
CFNB	2	206	0.672	0.86 (−3.13,4.86)	0.281 (14.1)	Fixed
Spinal anesthesia	2	275	0.000	−2.94 (−4.55,−1.33)	0.545 (0.0)	Fixed
General anesthesia	2	206	0.672	0.86 (−3.13,4.86)	0.281 (14.1)	Fixed

*SFNB* single femoral nerve block, *CFNB* continuous femoral nerve block, *VAS* visual analogue scale, *MD* mean difference

### Trial sequential analysis (TSA)

**Fig. 6:**
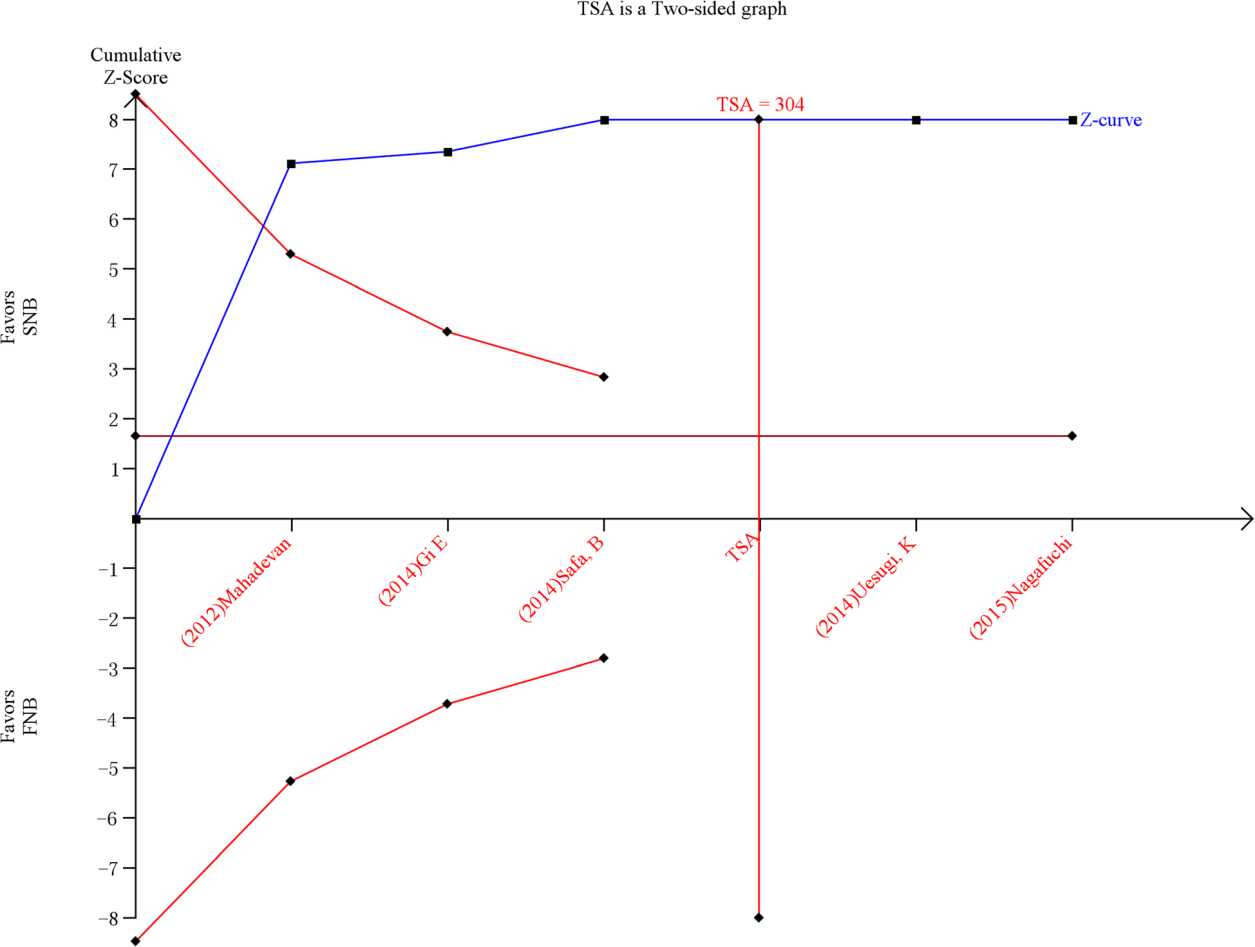
The trial sequence analysis of the visual analogue scale (VAS) scores with rest at 12 h, showing that the accumulative Z-curve crossed the trial sequential monitoring boundary for harm and surpassed the required information size

The trial sequential analysis results indicated that there was no need to perform additional studies to further identify the effects of SNB versus LIA for pain control after TKA. The cumulative *Z*-score crossed the *Z*-cure but did not reach the TSA value (Fig. 6).

### Active knee flexion

**Fig. 7:**
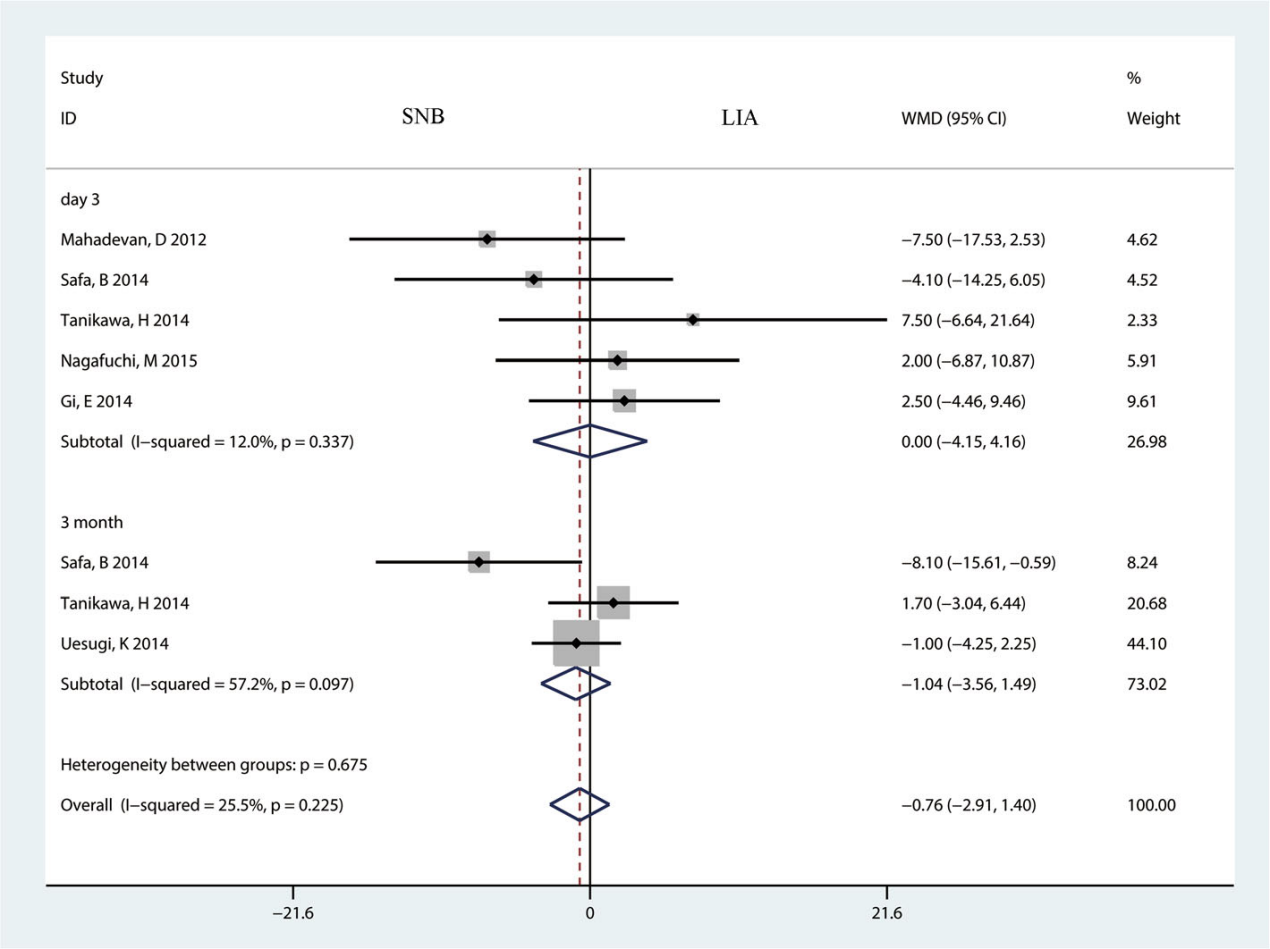
The forest plot comparing sciatic nerve block (SNB) and local infiltration anesthesia (LIA) for active knee flexion after TKA. An inverse variance fixed-effects model was used. Mean differences with 95% CIs are reported

A total of five studies with 245 patients and three studies with 321 patients reported active knee flexion in the SNB group and the LIA group on day 3 and month 3, respectively. The pooled results indicate that there was no significant difference between SNB and LIA in terms of active knee flexion on day 3 (MD = 0.00; 95% CI −4.15 to 4.16; *P* = 0.999, Fig. 7) or month 3 (MD = −1.40; 95% CI −3.56 to 1.49; *P* = 0.421, Fig. 7).

### Morphine consumption at 24 and 48 h

**Fig. 8:**
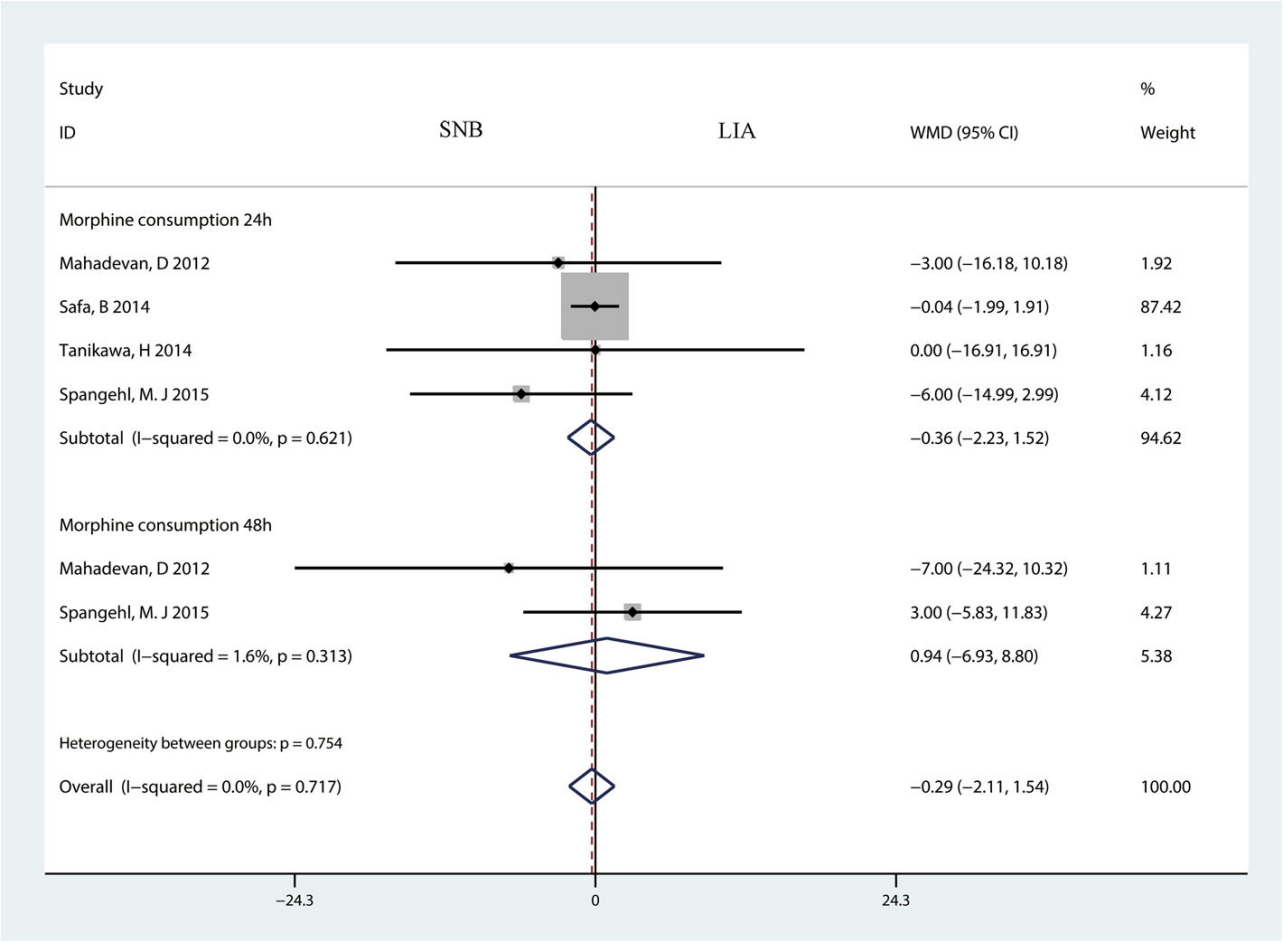
The forest plot comparing sciatic nerve block (SNB) and placebo in terms of morphine consumption at 24 and 48 h after TKA. An inverse variance fixed-effects model was used. Mean differences with 95% CIs are reported. *WMD* weighted mean difference

Four studies with 323 patients and two studies with 212 patients reported the morphine consumption at 24 and 48 h, respectively. The pooled results indicated that there was no significant difference between morphine consumption at 24 h (MD = −0.36; 95% CI −2.23 to 1.52; *P* = 0.708, Fig. 8) and 48 h (MD = 0.94; 95% CI −6.93 to 8.80; *P* = 0.816, Fig. 8) between the SNB group and the LIA group.

### Length of hospital stay

**Fig. 9:**
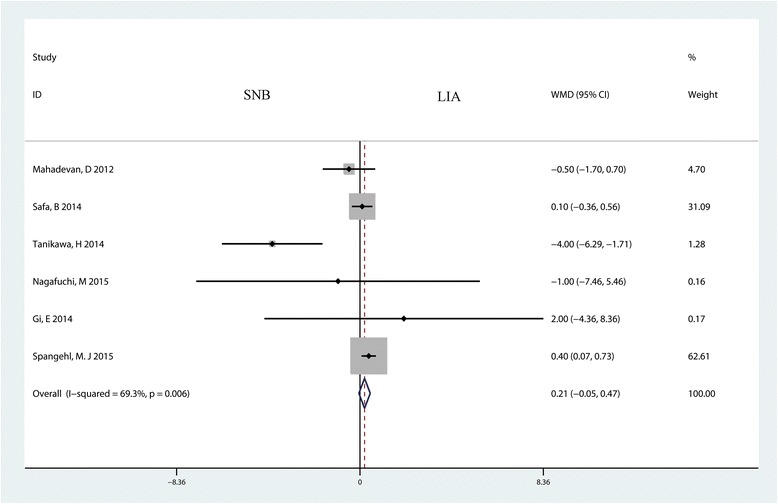
Forest plot comparing the length of hospital stay between the SNB group and the LIA group. *WMD* weighted mean difference

Six studies with 405 patients reported the length of hospital stay for the two groups. The result indicated that there was no significant difference between the SNB and LIA groups in terms of the length of hospital stay (MD = 0.21; 95% CI −0.05 to 0.47; *P* = 0.115, Fig. 9), and heterogeneity was moderate (*P* = 0.006, I^2^ = 69.3%).

### PONV

**Fig. 10:**
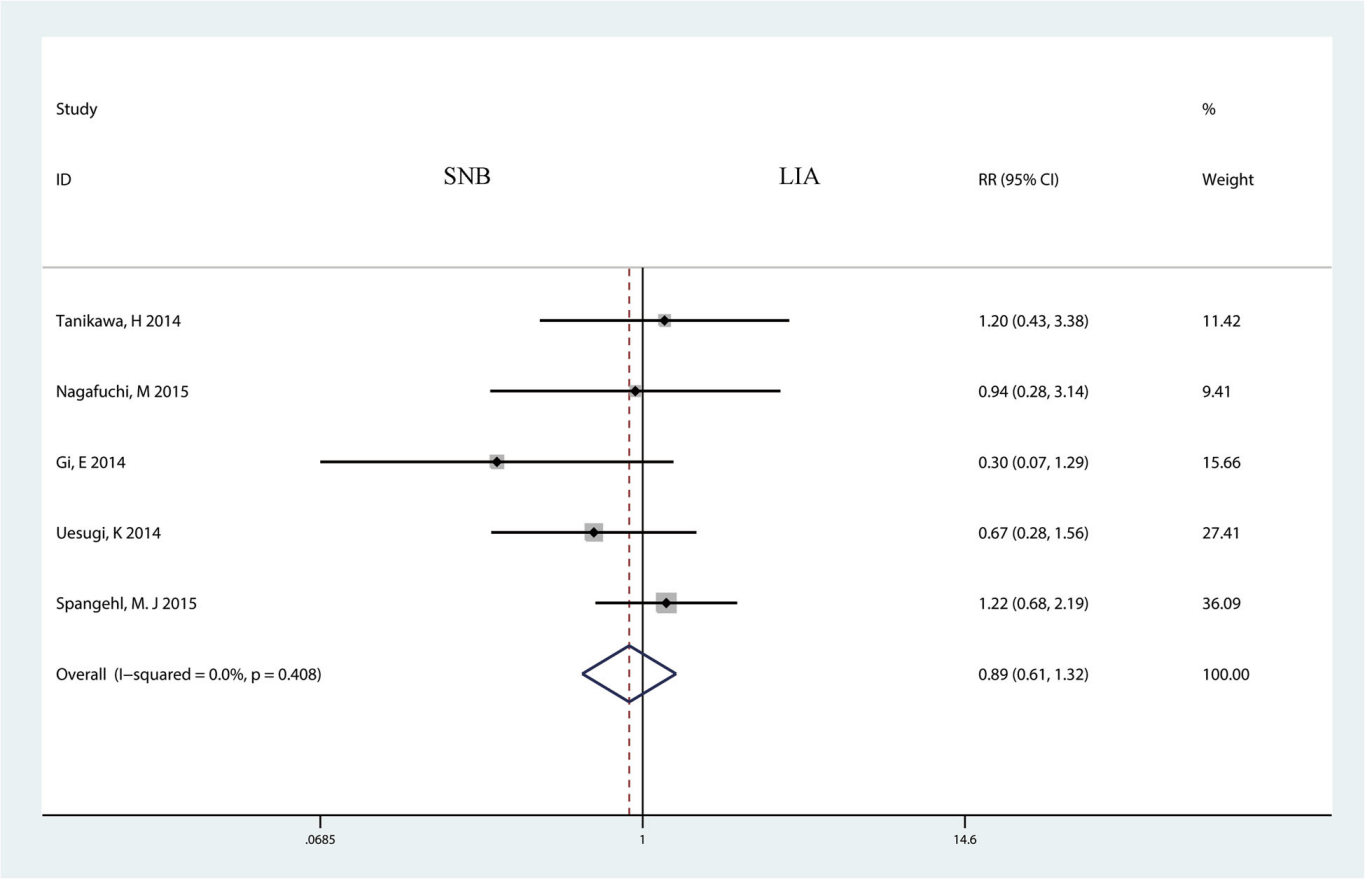
Forest plot comparing PONV between the SNB group and the LIA group. *RR* risk ratio

Six trials with 498 patients reported the occurrence of PONV for the two groups. The meta-analysis indicated that there was no statistically significant difference between the SNB and LIA groups (RR = 0.89, 95% CI 0.61 to 1.32, *P* = 0.575, Fig. 10).

### Falls

**Fig. 11:**
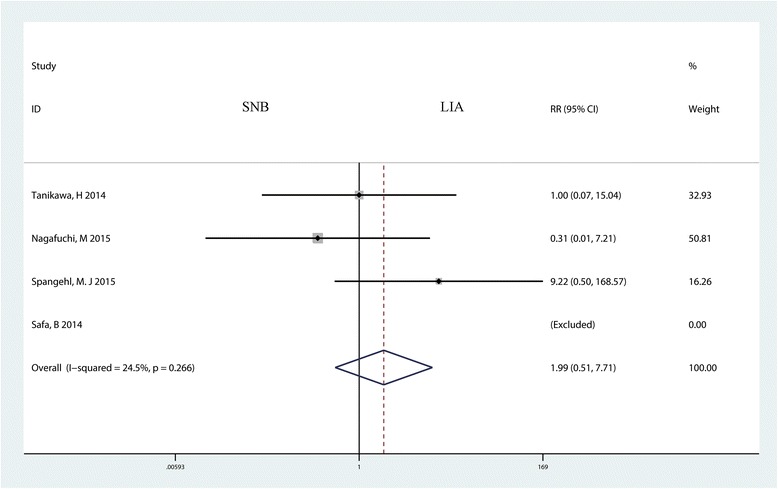
Forest plot comparing the occurrence of fall between the two groups. Mean differences with 95% CIs are reported. *RR* risk ratio

Four trials with 304 patients reported the occurrence of falls for the two groups. The meta-analysis indicated that there was no statistically significant difference between the SNB and LIA groups (RR = 1.99, 95% CI 0.51 to 7.71, *P* = 0.320, Fig. 11) in terms of the occurrence of falls.

### Anesthesia time

**Fig. 12:**
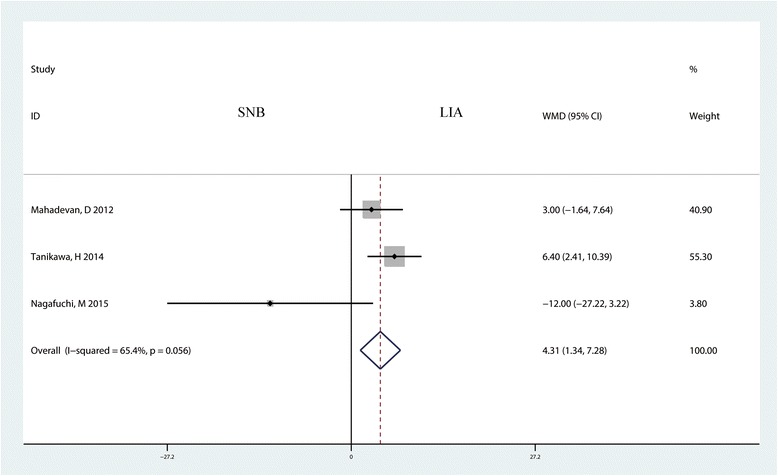
Forest plot comparing the anesthesia times of the two groups. Mean difference with 95% CIs are reported. *WMD* weighted mean difference

Three studies with 110 patients reported the anesthesia time for the LIA group and the SNB group. The pooled results indicated that the anesthesia time of the LIA group was shorter than that of the SNB group, and the difference was statistically significant (MD = 4.31, 95% CI 1.34 to 7.28, *P* = 0.004, Fig. 12).

## Discussion

This is the first systematic review and meta-analysis to compare combined femoral and SNB versus combined femoral with LIA for pain control after TKA. On the basis of the pooled estimates, combined femoral and SNB, compared with combined femoral with LIA, was associated with a reduction in pain scores, equivalent on an 110-point VAS to 6.96 point (95% CI, −8.36 to −5.56 point) at 12 h and 2.41 point (95% CI, −3.90 to −0.91) at 48 h. However, this reduction is not of clinical importance. There was no significant difference between active knee flexion, morphine consumption at 24 and 48 h, length of hospital stay, and the occurrence of PONV. And combined femoral and LIA, compared with combined femoral with SNB, are associated with less anesthesia time.

The only positive result is that SNB can decrease the VAS score at 12 and 48 h compared with LIA, and the difference is statistically significant. These outcomes concur with the morphine consumption at 24 and 48 h. Peripheral nerve block was induced before surgery and can decrease the morphine consumption during the early period. Morphine consumption was also used as a marker to test the efficacy of adjunctive analgesia [25–27]. Abdallah et al. [12] conducted a systematic review, and the results indicated that there was insufficient evidence to support the effect of adding SNB to FNB for anesthesia following TKA. Meanwhile, there was no statistically significant difference between the morphine-related complications of PONV.

Regarding active knee flexion, there was no significant difference between the two groups on day 3 and month 3 after TKA. The present results did not find any significant difference in the progress of rehabilitation, knee mobilization, and length of hospital stay. These results also indicated that improved early anesthesia cannot facilitate early rehabilitation. The results are consistent with past reports that concluded that SNB or LIA had no benefit in terms of knee function or length of hospital stay [23, 28, 29].

It has been reported that the rate of peripheral nerve injury is 2.9/10,000 for FNB and 2.4/10,000 for SNB, and the incidence of permanent nerve damage is 1.5/10,000 [30]. Sciatic nerve injury is also a generally known complication after TKA, with an incidence of 1.3 to 2.2% [31, 32]. Thus, LIA is a relatively safe anesthetic technique for pain control after TKA. The occurrence of falls did not differ significantly between the two groups after TKA. Spanghel et al. [22] found that four patients in the SNB group and no patients in the LIA group fell after TKA, and one patient suffered from a lumbar vertebral fracture after a fall. Tanikawa et al. [18] reported that one patient in the LIA group and one in the SNB group fell after TKA. The patients in each group were not permitted to ambulate without assistance from nurses or physiotherapists to prevent falls. Furthermore, the duration of the motor block of toe motion in the LIA group was less than that in the SNB group, and the difference was statistically significant. Nagafuchi et al. [20] found that one patient in the LIA group fell after surgery. Although the results of this meta-analysis indicated that there was no significant difference between SNB and LIA in terms of fall, a larger sample may have shown a trend toward more falls among patients who underwent FNB combined with SNB.

There were several limitations to this meta-analysis: (1) only seven RCTs were included, and the sample sizes in each trial were not large, which could affect the final results; (2) the duration of follow-up in some studies was unclear, and long-term follow-up was necessary for this analysis; (3) the publication bias that existed in the meta-analysis also influenced the results; (4) variations in the methods for obtaining, preparing, and applying the perioperative anesthetic protocol currently constitute a limitation to performing any comparisons between studies; (5) there is gross variability in SNB and FNB techniques (single-shot or continuous), location and needle approach and in the concentration, and volume and frequency of local anesthetic administered; (6) no studies applied the postoperative recovery scale, and high-quality studies that include a multi-domain quality of recovery assessment tool are preferred.

## Conclusions

In conclusion, there were no differences in pain scores with rest at 24 h, morphine consumption, active knee flexion, the occurrence of fall, and PONV between the groups. Although some analgesic efficacy at 12 and 48 h were seen with the use of combined femoral and SNB, these were unlikely to be of clinical importance. LIA may offer a practical and potentially safer alternative to SNB. Further high-quality RCTs are needed to identify the optimal dose of LIA for reducing pain after TKA.

## Additional files

Additional file 1: The search strategy of the included studies. (DOCX 14 kb)
Additional file 2: The comparison results of the general characteristic. (DOCX 16 kb)
Additional file 3: The results of VAS with rest at 12 h, 24 h and 48 h after excluded the study of Safa B. (TIF 3014 kb)

## Abbreviations

FNB: Femoral nerve block
LIA: Local infiltration anesthesia
MD: Mean difference
PONV: Postoperative nausea and vomiting
RCTs: Randomized controlled trials
RR: Relative risk
SNB: Sciatic nerve block
TKA: Total knee arthroplasty
TSA: Trial sequential analysis
VAS: Visual analogue scale.
