# Supplementary material for: Combined femoral and sciatic nerve block versus femoral and local infiltration anesthesia for pain control after total knee arthroplasty: a meta-analysis of randomized controlled trials
Source: J Orthop Surg Res. 2016 Dec 7;11:158. doi: 10.1186/s13018-016-0495-6 (PMC5142141; doi:10.1186/s13018-016-0495-6)
Supplement: Additional file 1: — The search strategy of the included studies. (DOCX 14 kb) [file 13018_2016_495_MOESM1_ESM.docx]

Additional file 1: The search strategy of the included studies.

((((((LIA) OR "Anesthesia, Local"[Mesh]) OR local infiltration anesthesia)) AND ((SNB) OR sciatic nerve block))) AND ((((("Arthroplasty, Replacement, Knee"[Mesh]) OR TKR) OR TKA) OR total knee replacement) OR total knee arthroplasty).
