# Supplementary material for: Combined femoral and sciatic nerve block versus femoral and local infiltration anesthesia for pain control after total knee arthroplasty: a meta-analysis of randomized controlled trials
Source: J Orthop Surg Res. 2016 Dec 7;11:158. doi: 10.1186/s13018-016-0495-6 (PMC5142141; doi:10.1186/s13018-016-0495-6)
Supplement: Additional file 2: — The comparison results of the general characteristic. (DOCX 16 kb) [file 13018_2016_495_MOESM2_ESM.docx]

| Variables | Studies(n) | Patients(n) | P-value | Incidence | | |
| --- | --- | --- | --- | --- | --- | --- |
|  |  |  |  | MD(95%CI) | Heterogeneity P-value(I^2^) | Model |
| Age(year) | 4 | 334 | 0.922 | -0.07(-1.53,1.38) | 0.903(0.0) | Fixed |
|  |  |  |  |  |  |  |
| Sex(female) | 6 | 445 | 0.588 | 1.03(0.93,1.14) | 0.872(0.0) | Fixed |
|  |  |  |  |  |  |  |
| Weight(kg) | 3 | 282 | 0.429 | 1.07(-1.58,3.71) | 0.144(48.5) | Fixed |
|  |  |  |  |  |  |  |
| Surgery time(min) | 3 | 282 | 0.207 | -2.36(-6.02,1.30) | 0.402(0.0) | Fixed |
|  |  |  |  |  |  |  |

Additional file 2: The comparison results of the general characteristic.
