# Supplementary figures and images for: Combined femoral and sciatic nerve block versus femoral and local infiltration anesthesia for pain control after total knee arthroplasty: a meta-analysis of randomized controlled trials
Source: J Orthop Surg Res. 2016 Dec 7;11:158. doi: 10.1186/s13018-016-0495-6 (PMC5142141; doi:10.1186/s13018-016-0495-6)

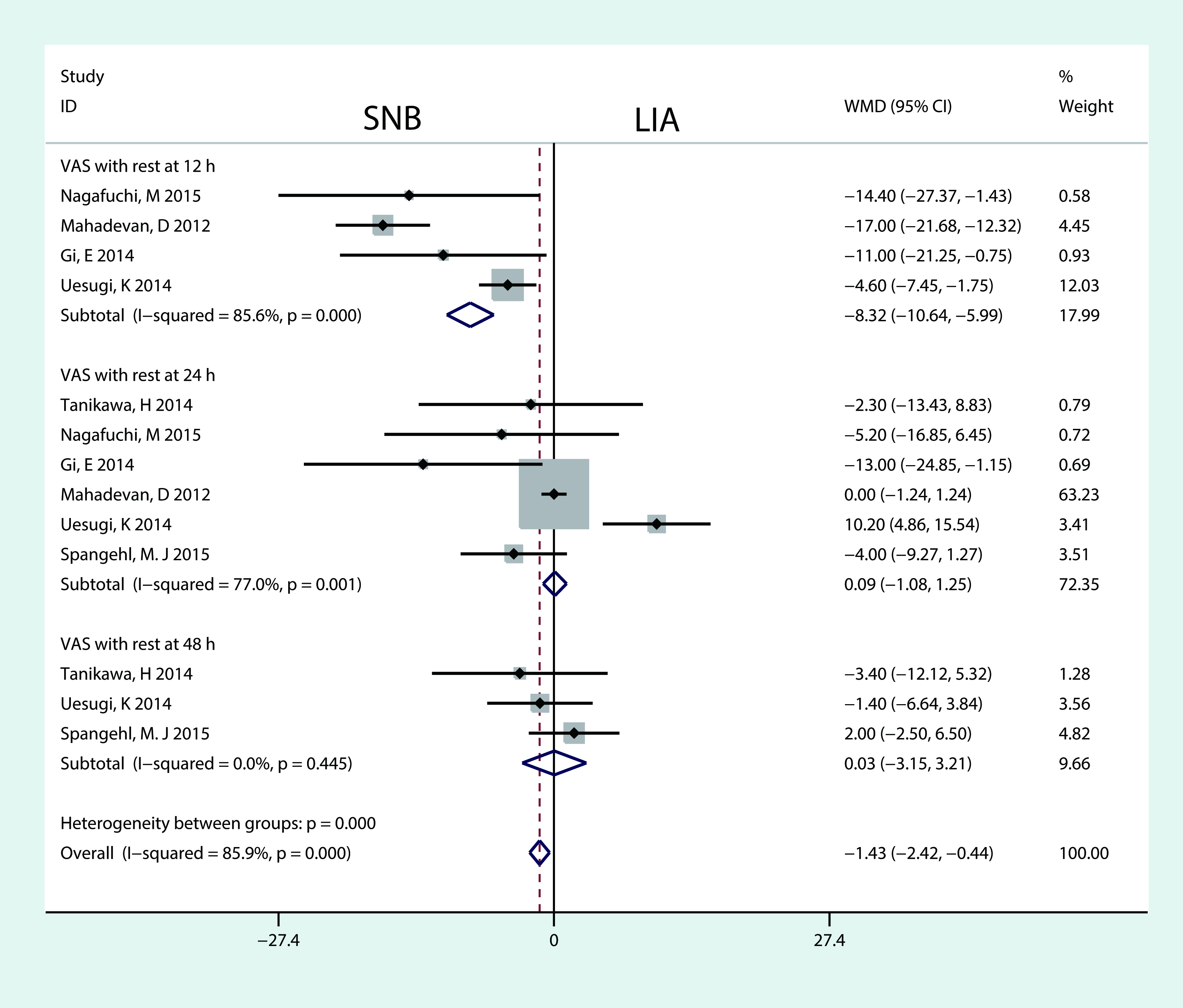

Supplement: Additional file 3: — The results of VAS with rest at 12 h, 24 h and 48 h after excluded the study of Safa B. (TIF 3014 kb) [file 13018_2016_495_MOESM3_ESM.tif]
